# Predicting response to immunotherapy in advanced non-small-cell lung cancer using tumor mutational burden radiomic biomarker

**DOI:** 10.1136/jitc-2020-000550

**Published:** 2020-07-06

**Authors:** Bingxi He, Di Dong, Yunlang She, Caicun Zhou, Mengjie Fang, Yongbei Zhu, Henghui Zhang, Zhipei Huang, Tao Jiang, Jie Tian, Chang Chen

**Affiliations:** 1 School of Electronic Electrical and Communication Engineering, University of the Chinese Academy of Sciences, Beijing, China; 2 CAS Key Laboratory of Molecular Imaging, Institute of Automation, Chinese Academy of Sciences, Beijing, China; 3 School of Artificial Intelligence, University of the Chinese Academy of Sciences, Beijing, China; 4 Department of Thoracic Surgery, Tongji University Affiliated Shanghai Pulmonary Hospital, Shanghai, China; 5 Department of Medical Oncology, Tongji University Affiliated Shanghai Pulmonary Hospital, Shang hai, China; 6 Beijing Advanced Innovation Center for Big Data-Based Precision Medicine, School of Medicine and Engineering, Beihang University, Beijing, China; 7 Department of Medicine, Beijing Genecast Biotechnology Co, Beijing, China; 8 Engineering Research Center of Molecular and Neuro Imaging of Ministry of Education, School of Life Science and Technology, Xidian University, Xi'an, China; 9 Key Laboratory of Big Data-Based Precision Medicine (Beihang University), Ministry of Industry and Information Technology, Beijing, China

**Keywords:** immunotherapy, lung neoplasms, tumor microenvironment, biomarkers, tumor, biostatistics

## Abstract

**Background:**

Tumor mutational burden (TMB) is a significant predictor of immune checkpoint inhibitors (ICIs) efficacy. This study investigated the correlation between deep learning radiomic biomarker and TMB, including its predictive value for ICIs treatment response in patients with advanced non-small-cell lung cancer (NSCLC).

**Methods:**

CT images from 327 patients with TMB data (TMB median=6.067 mutations per megabase (range: 0 to 42.151)) were retrospectively collected and randomly divided into a training (n=236), validation (n=26), and test cohort (n=65). We used 3D-densenet to estimate the target tumor area, which used 1020 deep learning features to distinguish High-TMB from Low-TMB patients and establish the TMB radiomic biomarker (TMBRB). The TMBRB was developed in the training cohort combined with validation cohort and evaluated in the test cohort. The predictive value of TMBRB was assessed in a cohort of 123 NSCLC patients who had received ICIs (survival median=462 days (range: 16 to 1128)).

**Results:**

TMBRB discriminated between High-TMB and Low-TMB patients in the training cohort (area under the curve (AUC): 0.85, 95% CI: 0.84 to 0.87))and test cohort (AUC: 0.81, 95% CI: 0.77 to 0.85). In this study, the predictive value of TMBRB was better than that of a histological subtype (AUC of training cohort: 0.75, 95% CI: 0.72 to 0.77; AUC of test cohort: 0.71, 95% CI: 0.66 to 0.76) or Radiomic model (AUC of training cohort: 0.75, 95% CI: 0.72 to 0.77; AUC of test cohort: 0.74, 95% CI: 0.69 to 0.79). When predicting immunotherapy efficacy, TMBRB divided patients into a high- and low-risk group with distinctly different overall survival (OS; HR: 0.54, 95% CI: 0.31 to 0.95; p=0.030) and progression-free survival (PFS; HR: 1.78, 95% CI: 1.07 to 2.95; p=0.023). Moreover, TMBRB had a better predictive ability when combined with the Eastern Cooperative Oncology Group performance status (OS: p=0.007; PFS: p=0.003). Visual analysis revealed that tumor microenvironment was important for predicting TMB.

**Conclusion:**

By combining deep learning technology and CT images, we developed an individual non-invasive biomarker that could distinguish High-TMB from Low-TMB, which might inform decisions on the use of ICIs in patients with advanced NSCLC.

## Introduction

Lung cancer is the leading cause of cancer-related mortality worldwide.^1^ Immune checkpoint inhibitors (ICIs), which target programmed cell death protein 1 (PD-1) and its ligand (PD-L1) or cytotoxic T lymphocyte antigen-4 (CTLA-4) can elicit durable antitumor responses in multiple cancer types, including non-small-cell lung cancer (NSCLC).^2–5^ Yet, only a minority of patients with advanced NSCLC derive clinical benefit from this treatment.^6^ Therefore, there is an urgent need to investigate the predictive biomarkers for ICIs treatment effectiveness.

With the development of next-generation sequencing, tumor mutational burden (TMB) has become a research focus. TMB has been suggested as capable of predicting the response to PD-1/PD-L1 blockade in patients with NSCLC.^7–10^ In addition, other potential biomarkers have been investigated, such as PD-L1 expression,^11–13^ tumor-infiltrating lymphocytes,^14 15^ specific gene mutations,^16–18^ and inflammatory cytokines.^19^ However, all these potential biomarkers require an invasive biopsy combined with time-consuming and labour-intensive laboratory and clinical testing. As such, a non-invasive biomarker to predict the efficacy of immunotherapy in NSCLC would be valuable.

In this context, radiomics has been proposed as a tool to quantitatively analyze tumor characteristics.^20^ This process involves dig deep features of tumors, which are not detected by the human eye and which have been suggested as a valuable aid in clinical diagnostics. Previous studies based on radiomics have yielded results relevant to auxiliary diagnosis,^21 22^ choice of treatment options,^23 24^ and assessment of patient prognosis.^25 26^ In addition, existing studies have confirmed that the CT images of tumors emerge significant differences during different ICIs therapy cycles through radiomics technology.^27^ With an increase in availability of and access to modern technologies, deep learning as a method for analyzing tumors has gained popularity among researchers. In fact, working with deep learning technologies has helped identify new research areas within the field of radiomics and integrate knowledge of tumor microenvironment in clinical analysis.

To our knowledge, there have been few previous attempts at quantitative imaging analyzes, using the deep learning approach in studies assessing predictive value of TMB in immunotherapy response.^28 29^ Moreover, only few previous studies focused on patients with NSCLC treated with ICIs.^27^ Given these considerations, the aim of the present study was to develop and validate a deep learning-based TMB radiomic biomarker (TMBRB), using CT images of patients with NSCLC. The secondary aim of this study was to assess the predictive value of TMBRB for clinical outcomes in patients with advanced NSCLC who had received ICIs treatment.

## Materials and methods

### Patients

Two data sets, TMB (n=327) and immunotherapy data set (n=123), were included in this study. The purpose of the TMB data set, randomly divided into training (n=236) validation (n=26) and test cohort (n=65), was to develop and validate TMRRB. It should be noted that the training cohort was used to identify the potential predictive value of CT images, the validation cohort was used to optimize the hyperparameters, and the test cohort was used to evaluate the TMBRB. The purpose of the immunotherapy data set was to assess the predictive value of TMRRB.

For the TMB data set, we retrospectively identified patients who had undergone complete resection of lung adenocarcinoma or squamous cell carcinoma at the Shanghai Pulmonary Hospital from 2012 to 2015. First, we checked the histological subtype of each patient using his or her electronic medical records. Subsequently, two experienced pathologists (ZWD and LKH) independently evaluated eligible specimens, according to the 2015 WHO classification of lung cancer. Major exclusion criteria were: inadequate or poor-quality samples, missing data on baseline clinicopathological features, mixed histology, and incomplete follow-up data. The details of the whole-exome sequencing and data processing are described in our previous study.^30^ The major baseline characteristics included in the TMB data set were age, sex, tumor stage, and tumor histology. Consistent with a previous study,^18^ we defined High-TMB as TMB value ≥10 mutations per megabase (mut/Mb), and Low-TMB as TMB <10 mut/Mb. These TMB cut-offs are commonly used, as they constitute a brief way of clarifying relevant investigations.

For the immunotherapy data set, data from patients diagnosed with advanced NSCLC who had received anti-PD-1/PD-L1 monotherapy at the Shanghai Pulmonary Hospital from 2015 to 2018 were collected. The major baseline characteristics included in this data set were age, sex, Eastern Cooperative Oncology Group performance status (ECOG PS), smoking history, tumor stage, tumor histology, Epidermal Growth Factor Receptor (EGFR) mutations, progression-free survival (PFS), and overall survival (OS). A never-smoker was defined as a patient who had smoked fewer than 100 cigarettes ahead of receiving systemic treatment. PFS was defined as the time from initial immunotherapy until disease progression or intolerable treatment toxicity. OS was calculated from the date of tumor diagnosis to death from any cause or was censored at the date of last follow-up. Clinical response to immunotherapy was evaluated based on the Response Evaluation Criteria in Solid Tumours (RECIST) V.1.1.^31^

### CT image and tumor segmentation

Patients whose CT images taken prior to treatment were unavailable were excluded from both data sets. The included CT images were obtained with scanners manufactured by Siemens (Somatom Definition AS+, Biograph64), Philips (Brilliance 40, iCT256, Ingenuity Flex, MX 16-slice), GE Medical System (Bright Speed), and United Imaging (uCT 510, uCT 760, uCT S-160). All images were reconstructed using slice thickness of 0.6, 1, 1.25, 2, 3, or 5 mm.

Two thoracic radiologists (TTW and YY) independently reviewed all scans. Assigned marks were determined by the consensus of the third thoracic radiologists (XWS). The region of interest annotation was performed with 3D slicer (http://www.slicer.org) for each lesion.^32^ The 3D centre-of-mass location was marked, and a bounding box was constructed to include the whole tumor.

Before training the model, we first normalized the CT images to eliminate radiographic differences between images acquired with different scanners. Afterwards, we generated new data by moving the bounding box marked by the doctors. This data augmentation operation provided new training samples for the model and helped reduce the errors introduced by the doctor’s annotation. Detailed data augmentation methods are described in online supplementary appendix 1.

10.1136/jitc-2020-000550.supp1 Supplementary data

### TMBRB construction and validation

**Figure 1:**
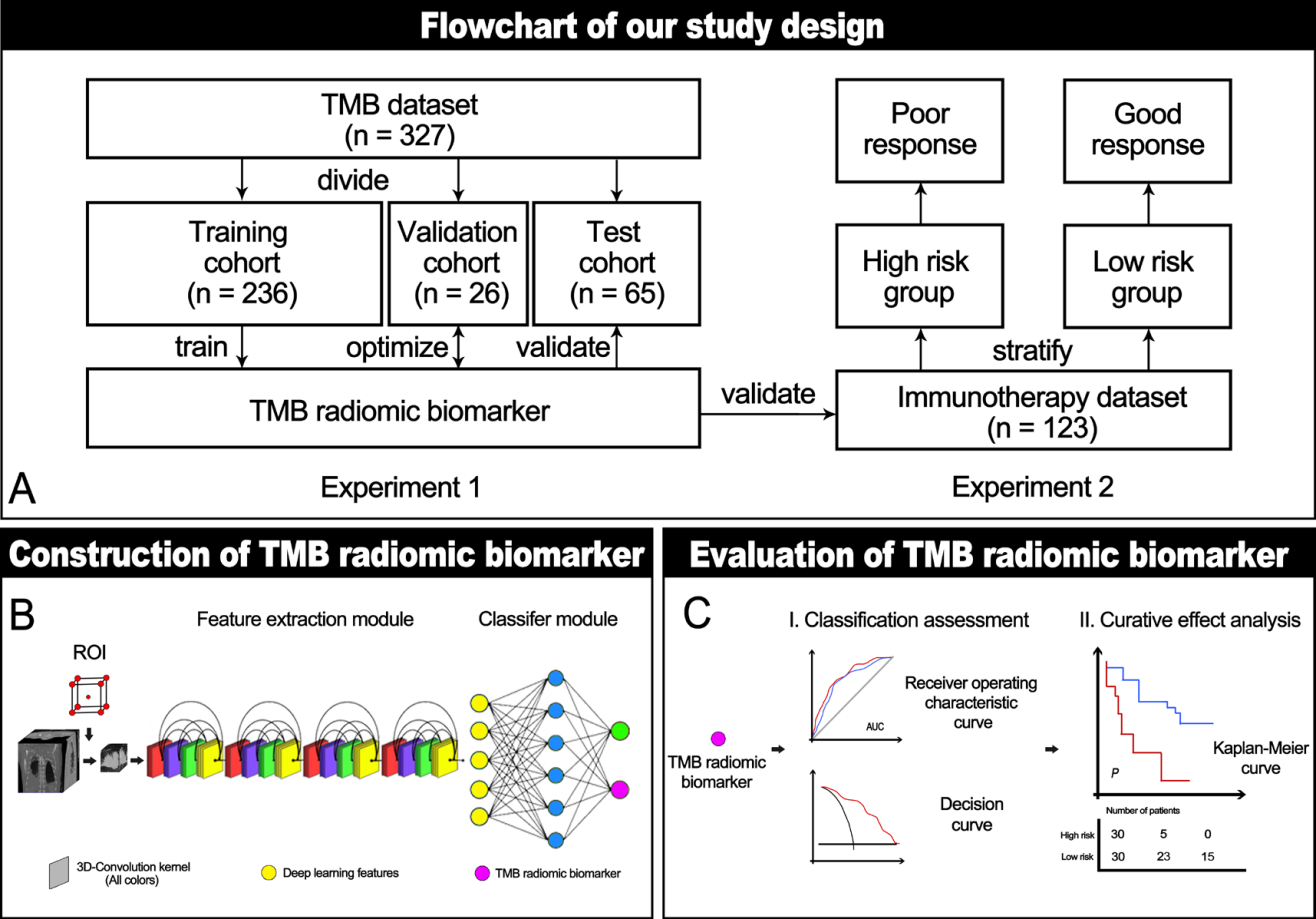
Study protocol workflow. (A) The main experiments in this study included: establishment and verification of TMBRB, and exploration of TMBRB value in predicting immunotherapy efficacy. (B) Structural diagram of the deep learning model. (C) The methods we used to evaluate TMBRB. AUC, area under the curve; ROI, region of interest; TMBRB, tumor mutational burden radiomic biomarker.

To generate TMBRB, we built a deep learning model (figure 1B). We used the patient's tumor area as training data to identify patients with High-TMB. In this study, TMBRB was defined as the model’s score for High-TMB output.

The deep learning model included two main modules: feature extraction and classification module. The input of the feature extraction module was the tumor region assessed based on the patient’s CT image. Structurally, it was mainly composed of Densenet with a 3D convolution kernel (3D-densenet).^33^ The module contained a total of four blocks, with dense connections within each block. This network structure could learn deeper information from the CT images, accelerate convergence, and, to a certain extent, avoid over-fitting. The output of this module included 1020 deep learning features.

For the classification module, we chose the fully connected network as the classifier, composed of an input layer, a hidden layer, and an output layer. The input layer of this module contained all the deep learning features. The hidden layer contained 128 nodes, and the output layer consisted of the patients’ High-TMB and Low-TMB scores. Details of network training configurations and training mode are described in online supplementary appendix 2.

10.1136/jitc-2020-000550.supp2 Supplementary data

For comparisons, we used ‘radiomics’ method to build the model (radiomic model), which quantitatively extracts predefined features from CT images. Details about the radiomic model is shown in online supplementary appendix 3. In addition, a clinical model was constructed employing the clinicopathological characteristics that were significantly related to the TMB level. Meanwhile, we also incorporated the comparative analysis of the maximum 3D-diameter and volume of the tumor.

10.1136/jitc-2020-000550.supp3 Supplementary data

The receiver operating characteristic curve (ROC) was used to evaluate the model’s ability to distinguish High-TMB from Low-TMB. The area under the curve (AUC), sensitivity, and specificity were calculated to compare performance between cohorts and models. The Delong test was used to compare the ROC between the models. In addition, a decision curve was drawn to quantify the net benefit under different threshold probabilities and evaluate the clinical utility of TMBRB. To further explore the potential of the biomarker, we verified its performance at different TMB cut-off points.

### The predictive value of TMRRB for immunotherapy

In the immunotherapy data set, TMBRB was applied to evaluate risk stratification at the individual level. All cut-off points for TMBRB were calculated by X-tile.^22^ For the evaluation method, we used the Kaplan-Meier curves to assess the OS and PFS. The log-rank test was used to assess different survival curves. In addition, the Cox proportional hazard model was used in multivariate analysis of TMBRB and clinicopathological characteristics. By comparing the significance of the model derived in multivariate analysis and the results of the survival curve comparison, we identified potential clinicopathological characteristics that could be combined with the biomarker to improve the model’s overall predictive value. TMBRB verification process is shown in figure 1C.

### Statistical analysis

Patients’ baseline characteristics were compared using Pearson χ^2^ test or Fisher’s exact test for categorical variables, when appropriate, and independent t-test for continuous variables. For the correlation study of TMB and TMBRB, we chose Spearman correlation coefficient as the evaluation standard. Furthermore, we performed all analyzes in R (V.3.5.2; http://www.R-project.org) and Python (V.3.6.5, https://www.python.org/). A two-sided p value<0.05 was regarded as statistically significant. The R and Python packages are summarized in online supplementary appendix 4.

10.1136/jitc-2020-000550.supp4 Supplementary data

## Results

### Clinicopathological characteristics of the cohorts

The clinicopathological characteristics of the TMB data set are summarized in online supplementary table S1. The number of patients in the training, validation, and test cohorts were 236, 26, and 65, respectively. In total, the sample of 327 patients included 180 (55.0%) men and 181 adenocarcinomas (55.4%), with median age of 61.5 years. The majority of them (74.3%, n=243) were classified into Low-TMB, with the remainder of the sample classified as High-TMB, with the overall mean value of 7.64 mut/Mb. No significant differences were observed between the training, validation, and test cohorts regarding age, sex, histological subtype, pathological stage, and TMB value. In the training cohort (online supplementary table S2), histological subtype was identified as a significant independent predictor of High-TMB (p<0.001). In the immunotherapy data set (table 1), most of them were men (82.1%) and current or former smokers (62.6%), with mean age of 61.8 years. The most common tumor pathological stage was stage IV (85.4%) and the most common histological type was adenocarcinoma (65.0%).

10.1136/jitc-2020-000550.supp5 Supplementary data

10.1136/jitc-2020-000550.supp6 Supplementary data

**Table 1:** Clinicopathological characteristics of the immunotherapy data set

Characteristics	All (n=123)	Overall survival			Progression-free survival		
		High-risk group (n=79)	Low-risk group (n=44)	P value	High-risk group (n=91)	Low-risk group (n=32)	P value
Age, year	61.8±10.2	61.5±10.9	62.4±8.7	0.646	62.5±10.7	60.0±8.2	0.247
Sex				0.424			0.512
Male	101 (82.1)	67 (84.8)	34 (77.3)		73 (80.2)	28 (87.5)	
Female	22 (17.9)	12 (15.2)	10 (22.7)		18 (19.8)	4 (12.5)	
Smoking status				0.685			0.533
Current or former smoker	77 (62.6)	51 (64.6)	26 (51.9)		55 (60.4)	22 (68.8)	
Never smoked	46 (37.4)	27 (40.9)	18 (40.9)		36 (39.6)	10 (31.2)	
ECOG performance-status score				0.364			0.993
0	11 (8.9)	7 (8.9)	4 (9.1)		8 (8.8)	3 (9.4)	
1	104 (84.6)	65 (82.3)	39 (88.6)		77 (84.6)	27 (84.4)	
2	8 (6.5)	7 (8.9)	1 (2.3)		6 (6.6)	2 (6.2)	
Tumor histologic type				0.728			0.247
Adenocarcinoma	80 (65.0)	50 (63.3)	30 (68.2)		56 (61.5)	24 (75.0)	
Squamous cell carcinoma	43 (35.0)	29 (36.7)	14 (31.8)		35 (38.5)	8 (25.0)	
Pathological stage				0.974			0.492
III	18 (14.6)	11 (13.9)	7 (15.9)		15 (16.5)	3 (9.4)	
IV	105 (85.4)	68 (86.1)	37 (84.1)		76 (83.5)	29 (90.6)	
EGFR mutation				0.618			0.983
No mutation	94 (76.4)	62 (78.5)	32 (72.7)		69 (75.8)	25 (78.1)	
Mutation	29 (23.6)	17 (21.5)	12 (27.3)		22 (24.2)	7 (21.9)	
TMBRB				**<0.001**			**<0.001**
Mean	0.55±0.16	0.46±0.13	0.71±0.05		0.62±0.10	0.34±0.11	
Range	0.07 to 0.83	0.07 to 0.61	0.61 to 0.83		0.46 to 0.83	0.07 to 0.46	

Categorical data are shown as numbers (%) and continuous data as mean±SD.

ECOG, Eastern Cooperative Oncology Group; EGFR, Epidermal Growth Factor Receptor; TMBRB, tumor mutational burden radiomic biomarker.

### TMBRB construction and validation

**Figure 2:**
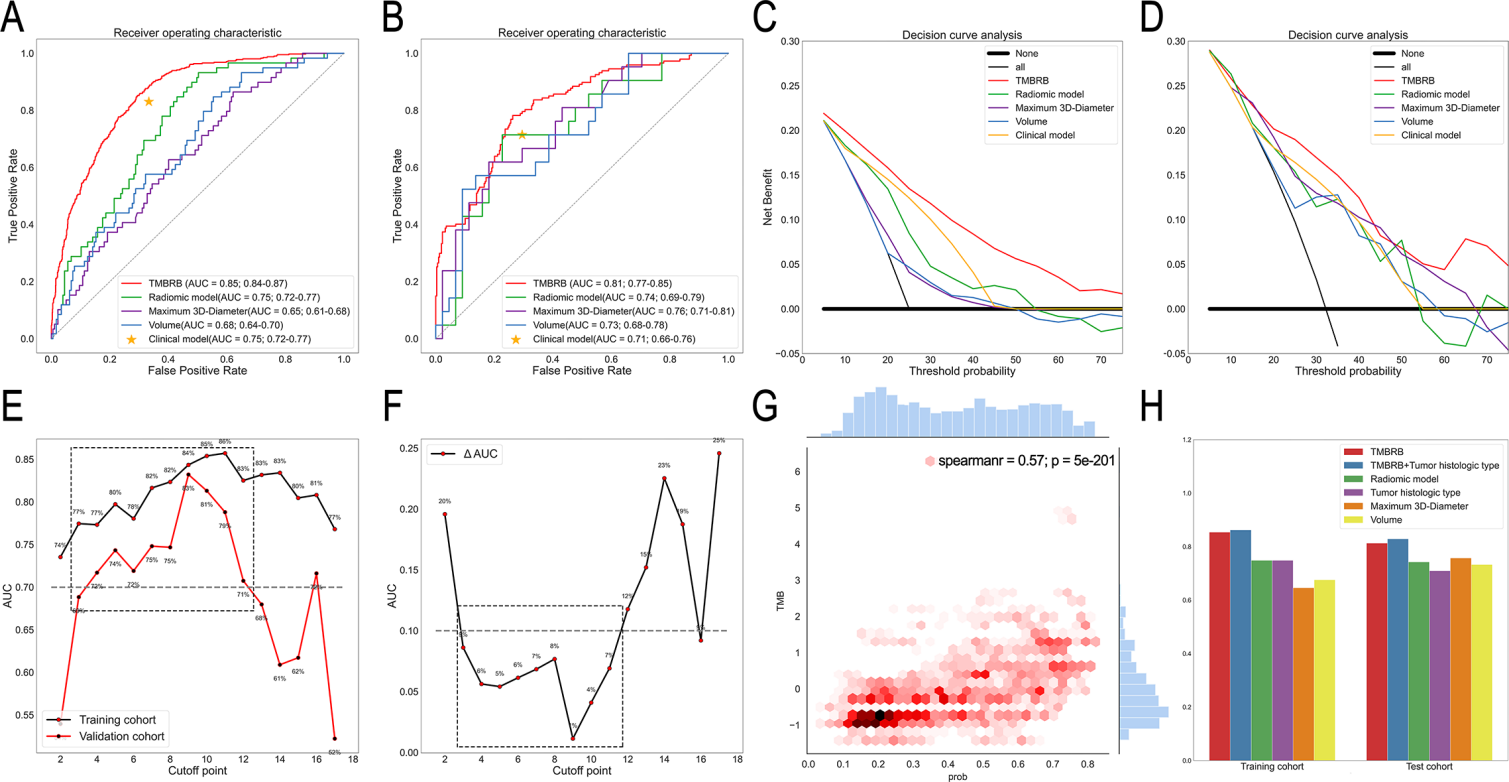
Ability of TMBRB to distinguish High-TMB from Low-TMB. (A) and (B) The receiver operating characteristic curve of the training and test cohorts based on TMBRB, radiomic model, clinical model, maximum 3D-diameter, and volume. (C) and (D) Decision curves for the training and test cohorts based on TMBRB, radiomic model, clinical model, maximum 3D-diameter, and volume. (E) and (F) AUC values for the training and test cohort with the TMBRB at different cut-off values of TMB and their difference line graphs; (G) Results of correlation analysis between TMB and TMBRB. (H) AUC values of different models in training and test cohorts. AUC, area under the curve; TMBRB, tumor mutational burden radiomic biomarker.

The AUC for TMBRB distinguishing between High- and Low-TMB groups was 0.85 (95% CI: 0.84 to 0.87) in the training cohort and 0.81 (95% CI: 0.77 to 0. 85) in the test cohort (figure 2A and B). In addition, although the radiomic model obtained good results in the training cohort (0.75; 95% CI: 0.72 to 0. 77) and the test cohort (0.74; 95% CI: 0.69 to 0.79), both were slightly lower than TMBRB. For the clinical model, since histological subtype was found significant, we used histological subtype to test the results in the training cohort (0.75; 95% CI: 0.72 to 0. 77) and test cohort (0.71; 95% CI: 0.66 to 0.76). We found that although the results of the training cohort and the test cohort are acceptable, they are not as good as TMBRB. This finding was confirmed by the decision curve. Overall, using TMRRB for decision-making emerged as a more robust approach compared with the radiomic or the clinical model (figure 2C and D). For the maximum 3D-diameter and volume, they did not yield a reliable and stable model. However, it is still necessary to discuss their impact on TMBRB. We conducted a Stratified analysis of patients according to the maximum 3D-diameter and volume. The results showed that TMBRB can perform better in different sizes and volumes (online supplementary figure S2B and S2C). In addition, we also found that the thickness did not affect TMBRB (online supplementary figure S2A). The details were shown in online supplementary appendix 5.

10.1136/jitc-2020-000550.supp7 Supplementary data

10.1136/jitc-2020-000550.supp8 Supplementary data

To excavate the potential ability of TMBRB, we used TMBRB and histological subtype for logistic regression. In the multivariate analysis, both p values of variables were less than 0.05 and the results were significantly improved in the training cohort (0.86; 95% CI: 0.84 to 0. 88; p<0.05) and the test cohort (0.83; 95% CI: 0.78 to 0. 87; p<0.05). For the relationship between histological subtypes and TMBRB, we can be sure that they will interact to improve the accuracy of prediction, but deeper interactions still need to incorporate more data for experimentation and verification.

Furthermore, we assessed the relationship between TMBRB and TMB in two ways. Since TMB is a continuous variable, we used the Spearman correlation coefficient for evaluation at first and the results showed that TMBRB and TMB have a strong correlation (correlation coefficient: 0.57; p<0.001; figure 2G). Moreover, we also checked the performance of TMBRB at different TMB cut-off, which can also clearly indicate the sensitivity distribution of TMBRB to TMB. As shown in figure 2E, TMBRB could effectively distinguish between two types of samples with cut-off points within the range 3 to 10 mut/Mb. The AUC for the 4 to 10 mut/Mb groups was each greater than 0.70 (including training cohort and test cohort), while the corresponding AUC differences between training and test cohorts were <0.10 (figure 2F). When the TMB cut-off value was 3 mut/Mb, the TMBRB exhibited good robustness, while the AUC of test cohort was about 0.69.

### Predictive value of TMBRB in the immunotherapy data set

**Figure 3:**
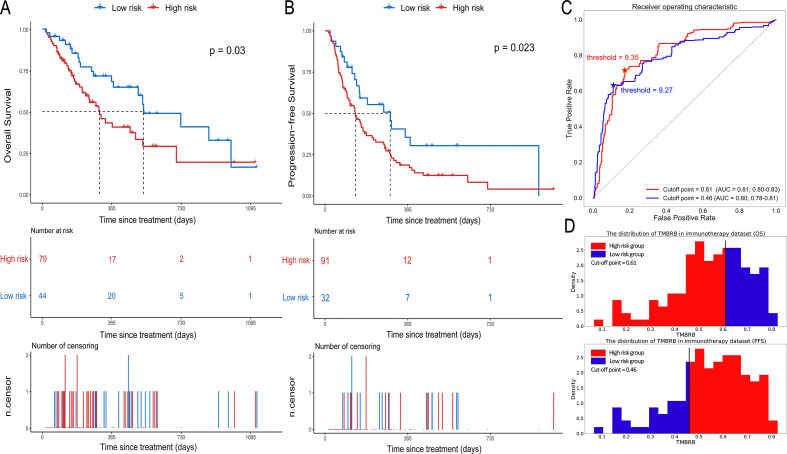
Prognostic value of TMBRB in immunotherapy. (A) and (B) The Kaplan-Meier curves depicting OS in high- and low-risk groups of OS and the high- and low-risk groups of PFS. (C) The receiver operating curves for TMB to distinguish between high- and low-risk groups and its best cut-off points (OS: 0.61; PFS: 0.46), which reflects the most likely cut-off points of TMB to be used for risk stratification (OS: 9.35; PFS: 9.27). (D) The distribution of TMBRB in immunotherapy data set, and the best cut-off points of TMBRB for OS and PFS. AUC, area under the curve; OS, overall survival; PFS, progression-free survival; TMBRB, tumor mutational burden radiomic biomarker.

TMBRB was able to divide patients into two risk cohorts (p=0.030; cut-off point=0.61; HR: 0.54, 95% CI: 0.31 to 0.95; figure 3A) with significantly different OS. In the dichotomy, the median OS was 301 days in the high-risk group and 533 days in the low-risk group. For PFS, TMBRB was also able to divide patients into two risk groups with better significant difference (p=0.023; cut-off point=0.46; HR: 1.78, 95% CI: 1.07 to 2.95; figure 3B). Moreover, the median PFS in the low-risk group was more than twice as much as the median PFS in the high-risk group (288 vs 134 days). Furthermore, the ROC curves for the high- and low-risk groups and TMB showed the optimal thresholds for TMB were 9.27 and 9.35, when the cut-off value was 0.46 and 0.61, respectively (figure 3D–F). We speculated that the best cut-off points of TMB for high- and low-risk stratification of NSCLC patients is between 9 and 10 for OS and PFS.

**Figure 4:**
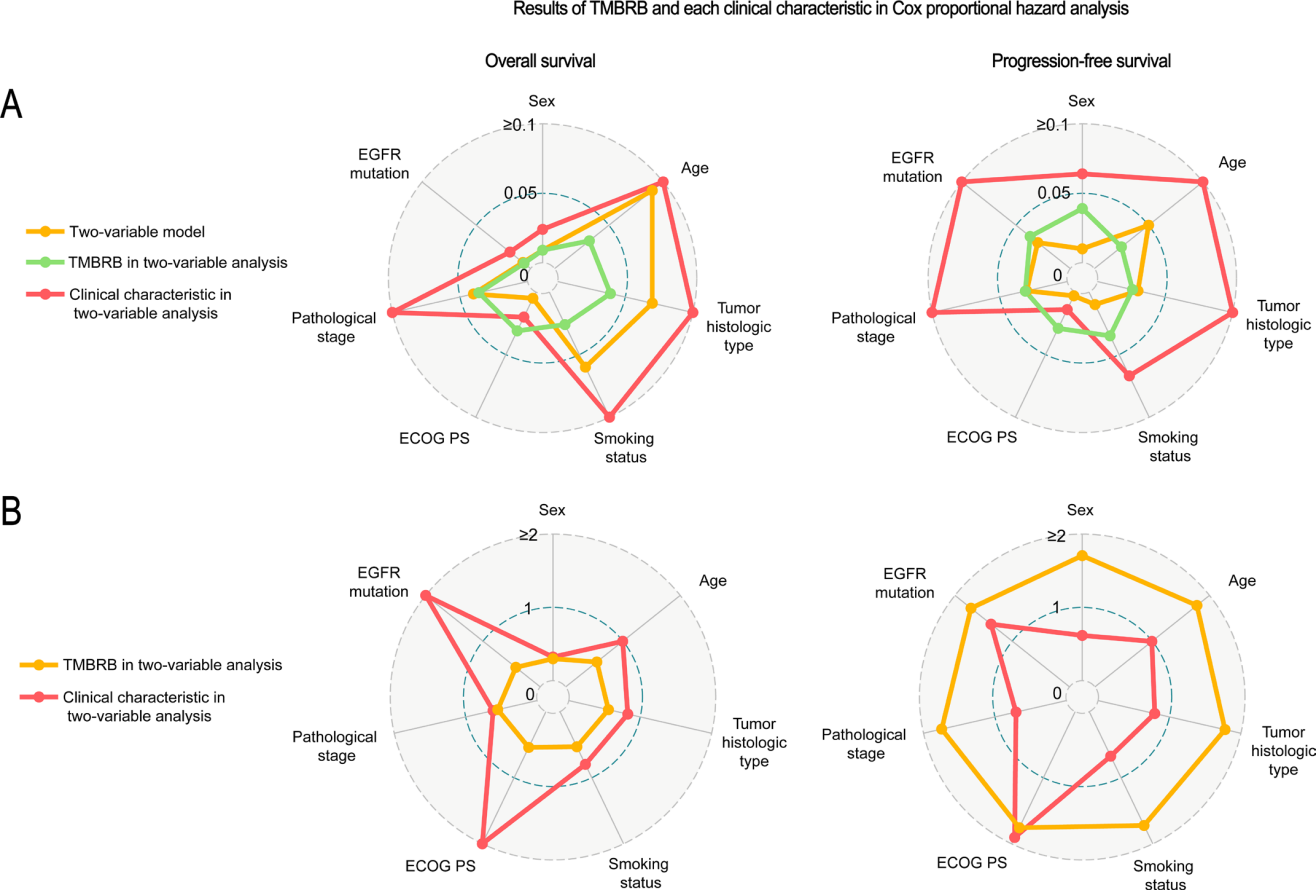
Clinicopathological characteristics associated with TMBRB in immunotherapy prediction. Evaluation of TMBRB and various clinicopathological characteristics. (A) The p value of each clinical characteristic and TMBRB in two-variable analysis by Cox regression and the log-rank p value of its model for OS and PFS. (B) Hazard rates of TMBRB and clinical characteristics in each two-variable model. ECOG PS, Eastern Cooperative Oncology Group performance status; OS, overall survival; PFS, progression-free survival; TMBRB, tumor mutational burden radiomic biomarker.

We recorded the results of univariate analysis of each clinical characteristic (online supplementary table S3) and the results of the log-rank test after combination of each characteristic and TMBRB for OS and PFS separately (figure 4A). We found that ECOG PS alone had a good stratification ability (OS: p=0.020; PFS: p=0.010). However, the combination of TMBRB and ECOG PS showed more significant stratification performance (OS: p=0.005; PFS: p=0.003). Figure 4A lists the p value for multivariate analysis after combination of each feature and TMBRB for OS and PFS, respectively. We found that the combination of ECOG PS and TMBRB improved the stratification results of the model and each variable remained statistically significant in the combined model. This situation appears not only in the analysis of OS (TMBRB: p=0.031; ECOG PS: p=0.020), but also in the analysis of PFS (TMBRB: p=0.029; ECOG PS: p=0.014). Figure 4B shows the hazard ratios of clinical characteristics and TMBRB binding for OS and PFS, respectively. In the model that included ECOG PS and TMBRB, for the analysis of OS, increase in the ECOG PS (HR: 2.33, 95% CI: 1.14 to 4.77) was associated with an increase patients’ risk; concurrently, an increase in TMBRB (HR: 0.54, 95% CI: 0.31 to 0.95) was associated with a decrease in patients’ risk. For PFS, the increase in TMBRB (HR: 1.76, 95% CI: 1.06 to 2.92) and ECOG PS (HR: 1.90, 95% CI: 1.14 to 3.19) are all associated with increased patient risk.

10.1136/jitc-2020-000550.supp9 Supplementary data

Excluding ECOG PS, we found that both sex (p=0.030) and smoking status (p=0.040) can stratify PFS risk very well. However, when combined with TMBRB analysis, sex (p=0.064), and smoking status (p=0.067) are not significant. For OS, the combination of EGFR mutation and TMBRB can significantly improve the performance of the model (p<0.05). In the model, both factors are significant (EGFR mutation: p=0.019; TMBRB: p<0.01). Moreover, although sex cannot be used for risk stratification alone (p=0.100), it has significant performance in combination with TMBRB (TMBRB: p=0.009; sex: p=0.024). And, the overall forecast level also improved significantly (p=0.010).

### Visualization and analysis of deep learning features

**Figure 5:**
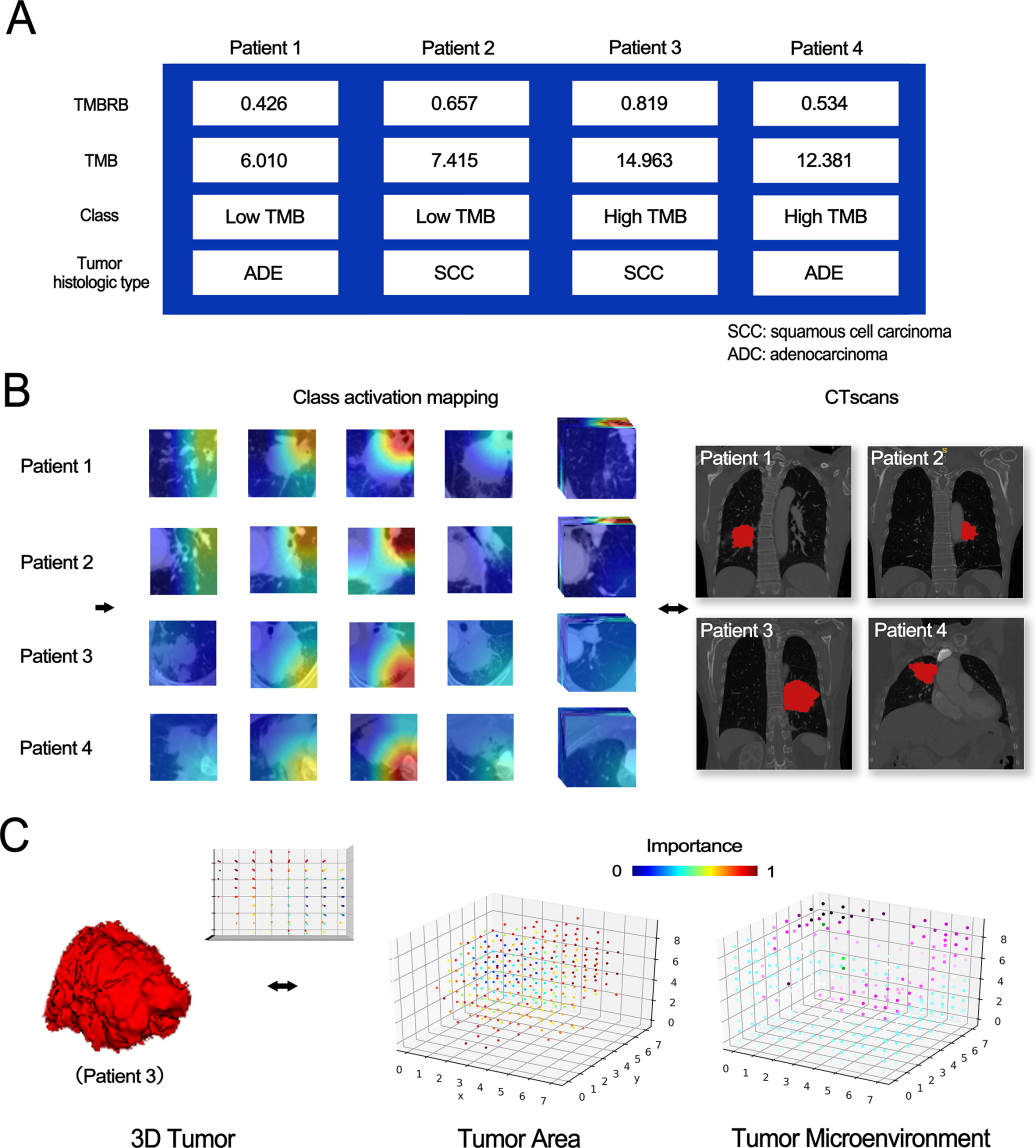
Visual analysis of TMBRB. Class activation maps for two types of samples and 3D visualization. (A) Clinical information of four patients. (B) The category activation map of TMBRB and the CT scans of patients. (C) The spatial lattice map of tumor and its microenvironment for Patient 3. TMB, tumor mutational burden; TMBRB, TMB radiomic biomarker.

To further examine the relationship between CT images and TMB, we exported the category activation map of TMBRB for research, as TMBRB had shown that it could effectively divide patients into high- and low-risk groups in the immunotherapy data set. The high importance area of TMBRB to some extent also covers areas that are important for improvement of OS to immunotherapy. The model diagram is shown in figure 5. For class activation maps, we found that, regardless of tumor histology type, the deep learning model paid more attention to the surrounding of the tumor and hilum. In addition, after resampling the class activation map and plotting the 3D space lattice, as well as separating the tumor and non-tumor regions, we found that the tumor microenvironment contributed no less to accuracy of prediction than did tumor region.

## Discussion

In this study, we constructed the TMBRB based on deep learning approach and found it could effectively divide patients into High-TMB and Low-TMB, as well as predict outcomes of NSCLC patients treated with ICIs. In addition, we discovered that the ECOG PS and TMRRB are mutually reinforcing at predicting treatment efficacy in this group of patients. Immunotherapy targeting PD-1 and PD-L1 is considered a ‘breakthrough’ treatment for advanced NSCLC.^16–18 34^ Despite durable response and improved prognosis, anti-PD-1/PD-L1 antibodies benefit a minority of patients. How to select patients most likely to benefit from immunotherapy is the current leading challenge in the field. In this context, the present study suggests that TMB has the potential to identify patients most likely to benefit from treatment with anti-PD-1/PD-L1 antibodies.^19 34^ However, multiple tumor sampling, invasive tissue biopsy, poor sample quality, and high associated costs limit the clinical applications of TMB. Therefore, development of a non-invasive approach to TMB calculation is required.

Due to the limited amount of training samples, it is difficult to train and obtain a satisfactory TMB predictive model via a regression method. A recent clinical trial reported that a TMB of at least 10 mut/Mb was an effective cut-off for predicting efficacy of immunotherapy.^18^ To simplify this model, we converted the TMB regression problem to a classification problem of High- and Low-TMB with a 10 mut/Mb cut-off. In addition, we not only found that TMBRB and TMB had a significant correlation via the Spearman correlation coefficient (correlation coefficient: 0.57; p<0.001), but also performed a classification assessment of TMBRB at different TMB cut-off points and found that TMBRB exhibited good classification performance within the TMB cut-off range of 3 to 11 mut/Mb. We infer that this was related to the distribution of the TMB data, as we observed that the closer to the median, the better the classification performance. In the case of a small amount of data, the classification problem makes the model more targeted than processing all TMB data for regression. Meanwhile, the distribution of some data will be ignored. As shown in the present study, the TMBRB can only distinguish between samples with TMB cut-offs range of 3 to 11 mut/Mb. For samples with TMB >11 mut/Mb, the classification ability of the model is insufficient.

In this study, we further validated the predictive value of TMBRB in an ICI-treated cohort. To the best of our knowledge, this is the first study to investigate the radiomic biomarker for TMB prediction in patients with advanced NSCLC. A previous study has shown that quantified radiomics features of lesions might function as non-invasive biomarkers for immunotherapy response, following analysis of 1055 primary and metastatic lesions from 203 patients with advanced melanoma and NSCLC.^29^ These imaging features were associated with pathways involved in mitosis, indicating a relationship between preferential response to immunotherapy and increased proliferative potential of a tumor. Similarly, our results revealed that, using a deep learning neural network, TMBRB could capture some high-level imaging features related to immunotherapy response and high-level TMB. Regardless of radiomics or deep learning technology, their advantage is not only that the data is easy to obtain, but also non-invasive for patients. Therefore, we believe our results offer evidence in support of a new non-invasive approach to survival evaluation in immunotherapy.

A previous study has indicated that radiomic signature of tumor-infiltrating CD8 cell was promising at predicting the immune phenotype of tumors and associated clinical outcomes in patients with NSCLC receiving anti-PD-1/PD-L1 treatment.^29^ In this study, all features, including technical variables, volume of interest locations, radiomic features of tumor, and peripheral rings were included in a linear elastic-net model. Moreover, the final radiomics signature included eight features, three of which represented the peripheral ring of the tumor. These findings are similar to our results, indicating that the peripheral tumor area is an important factor in determining TMBRB and immunotherapy response, and there may be a correlation between the peritumoral image and the abundance of CD8 cells. Meanwhile, Khorrami *et al* reported that there is a very important value in the ICIs therapy in the peritumoral period.^27^ One of the findings of the study is that the changes in a radiomic texture (DelRADx) feature named Haralick entropy shows significant differences in ICIs therapy. Moreover, this study also found a significant correlation between tumor-infiltrating lymphocyte (TIL) density and the peritumoral Gabor filter DelRADx feature. In the present study, we generated a class activation map to visualize TMBRB. We found that the area of interest of TMBRB to distinguish TMB is within as well as outside the tumor, concentrated at the tumor’s root and periphery. These results were similar to previous research, which maintains a high degree of attention to the peritumoral area.^27 29^ We also speculated that the area of interest of TMBRB is probably related to CD8 cell abundance and TIL density. Meanwhile, this area may be a relatively important location in the peritumoral area.

Our study had several limitations that should be acknowledged. First, this was a retrospective study based at a single medical center, including only Chinese patients. Selection bias was inevitable and whether the present findings apply to other ethnicities remains unknown. To be confirmed, the present findings require a multi-center, prospective study with a large, multi-ethnic sample. Second, TMBRB was constructed and validated using a cohort of patients with early-stage NSCLC. Its value in distinguishing TMB levels among advanced-stage NSCLC patients needs further investigation. Besides, since the number of patients with immunotherapy information has just exceeded 100, we have not divided an independent test set. For the effect of characteristics such as ECOG PS on TMBRB, we only speculated based on existing statistical results. In subsequent studies, we will include more patients for verification. Finally, recent studies revealed that several specific gene alterations (such as KEAP1, STK11, KRAS, among others.) could affect the efficacy of immunotherapy in NSCLC. Due to lack of sequencing data, we could not account for their role in determining TMB level and immunotherapy efficacy.

## Conclusion

In conclusion, our study indicated that deep learning could be a non-invasive method to evaluate TMB. The imaging biomarker derived from TMB could effectively predict clinical outcomes associated with ICIs treatment in patients with advanced NSCLC.

10.1136/jitc-2020-000550.supp10 Supplementary data
