# Supplementary material for: Predicting response to immunotherapy in advanced non-small-cell lung cancer using tumor mutational burden radiomic biomarker
Source: J Immunother Cancer. 2020 Jul 6;8(2):e000550. doi: 10.1136/jitc-2020-000550 (PMC7342823; doi:10.1136/jitc-2020-000550)
Supplement: Supplementary data [file jitc-2020-000550supp001.pdf]

Appendix S1: The method of data augmentation

When the radiologists selected the bounding-box of the tumor, there will inevitably be deviations. This deviation exists not only between the tumors, but also when the same tumor was marked at different times.

Therefore, we can amplify the data by changing the position of the bounding box, which not only eliminates the influence on the radiologists' mark as much as possible, but also makes the model focus more on the inside of the tumor. Besides, in the case of a small sample size, the model is easy to overfit.

The flow chart of data augmentation was shown in **FigureS1**. We move the bounding box by two pixels in the 2 directions of x-axis, y-axis, and z-axis, respectively. In this study, the number of samples of training cohort and validation cohort was amplified from 262 and 65 to 1834 and 455.

Figure S1. **The flow chart of data augmentation**
