# Supplementary material for: Predicting response to immunotherapy in advanced non-small-cell lung cancer using tumor mutational burden radiomic biomarker
Source: J Immunother Cancer. 2020 Jul 6;8(2):e000550. doi: 10.1136/jitc-2020-000550 (PMC7342823; doi:10.1136/jitc-2020-000550)
Supplement: Supplementary data [file jitc-2020-000550supp002.pdf]

Appendix S2: The configuration of deep learning model

Throughout the training process is divided into three steps:

1. we trained 3D-densenet and only used linear classifiers for the classification module.
2. we freeze the parameters of 3D-densenet to learn the full connection layer.
3. we used a small learning rate to fine tune the model.

In this study, the model was trained for 60 epochs (20*3) and we used a total of four learning rates (1e-4, 1e-5, 1e-6, and 1e-7), which were employed in sequence per 5 epochs. During the model training, we chose the loss of three consecutive epochs in validation cohort without dropping as the stopping criterion. While for the loss function, we used the cross-entropy loss function and the "Adam" was selected as our optimizer. We ran the deep learning program on 2 Nvidia TITAN XP GPUs with a total batch size of 64 and calculated program on the Inter(R) Core™ i7-8700K CPU @ 3.70GHz. The memory of the computer is 16G.
