# Supplementary material for: Predicting response to immunotherapy in advanced non-small-cell lung cancer using tumor mutational burden radiomic biomarker
Source: J Immunother Cancer. 2020 Jul 6;8(2):e000550. doi: 10.1136/jitc-2020-000550 (PMC7342823; doi:10.1136/jitc-2020-000550)
Supplement: Supplementary data [file jitc-2020-000550supp003.pdf]

Appendix S3: Construction of Radiomic model

The core of the Radiomic model is to describe the tumor area by manually pre-defined image features, and then build the model through the machine learning algorithm. Radiomics is not the same as deep learning in defining features.

We used python package named "pyradiomics" to extract the radiomic features [1]. The radiomic features are mainly divided into three categories, the first-order statistical feature, the morphological feature, and the texture feature. The first-order statistical feature is a feature obtained by statistically calculating the gray value of the area segmented by the doctor. The morphological features mainly describe the performance of tumors in two-dimensional and three-dimensional shape. The texture features mainly reflect the tumor internal texture information calculated by grey-level co-occurrence matrix (GLCM), Gray level run length matrix (GLRLM) and so on. These radiomic features which we extracted comply with feature definitions as described by the Imaging Biomarker Standardization Initiative (IBSI) [2].

In this experiment, we extracted a total of 1688 features. According to statistics, we employed 11 filters (including linear and nonlinear filters) for image transformation, and a total of 324 first-order statistical features, 14 morphological features, and 1350 texture features are predefined. The fully connected network which contains an input layer (1688 nodes), a hidden layer (128 nodes) and an output layer (2 nodes) was selected as our classifier. Meanwhile, we also added the batch normalization layer and weight decay to enhance the generalization ability of the model. In the model building process, we also used the training cohort to build the radiomic model, the validation cohort to adjust the parameters, and the test cohort to evaluate the model.

[1] Griethuysen, J. J. M. V. , Fedorov, A. , Parmar, C. , Hosny, A. , & Aerts, H. J. W. L. . (2017). Computational radiomics system to decode the radiographic phenotype. *Cancer Research, 77*(21), e104-e107.

[2] Zwanenburg A, Leger S, Vallières M, Löck SJapa (2016) Image biomarker standardisation initiative.
