# Supplementary material for: Predicting response to immunotherapy in advanced non-small-cell lung cancer using tumor mutational burden radiomic biomarker
Source: J Immunother Cancer. 2020 Jul 6;8(2):e000550. doi: 10.1136/jitc-2020-000550 (PMC7342823; doi:10.1136/jitc-2020-000550)
Supplement: Supplementary data [file jitc-2020-000550supp004.pdf]

Appendix S4: Packages

For Python packages, the Mann-Whitney U test we calculated by python package named “stats”. For the construction and training of deep learning models, we used the deep learning tensor library named “pytorch” (version 1.3; https://pytorch.org/). The AUC is calculated by the python package named “scikit-learn” (version 0.21.3; https://scikit-learn.org/stable/). For the multivariate analysis of clinical characteristics and TMB radiomic biomarker, we used python package named "lifelines" (version 0.22.10; https://lifelines.readthedocs.io/en/latest/) for calculation. For Python packages, the decision curve was calculated via the R package named "decisionCurve". We drew Kaplan-Meier curves by the R packages named ‘survival’ and ‘survminer’.
