# Supplementary material for: Predicting response to immunotherapy in advanced non-small-cell lung cancer using tumor mutational burden radiomic biomarker
Source: J Immunother Cancer. 2020 Jul 6;8(2):e000550. doi: 10.1136/jitc-2020-000550 (PMC7342823; doi:10.1136/jitc-2020-000550)
Supplement: Supplementary data [file jitc-2020-000550supp005.pdf]

Table S1 Clinicopathological characteristics of the TMB dataset

characteristics		ALL (N=327)	Training cohort (N=236)	Validation cohort (N=26)	*p* Value*	Test cohort (N=65)	*P* Value**
Age, year		61.5 ± 9.0	61.6 ± 8.9	61.6 ± 9.1	0.976	61.6 ± 9.5	0.741
Sex					0.385		0.433
	Male	180 (55.0)	135 (57.2)	12 (46.2)		33 (50.8)	
	Female	147 (45.0)	101 (42.8.3)	14 (53.8)		32 (49.2)	
Histological subtype					0.615		0.807
	Adenocarcinoma	181 (55.4)	128 (54.2)	16 (61.5)		37 (56.9)	
	Squamous cell carcinoma	146 (44.6)	108 (45.8)	10 (38.5)		28 (43.1)	
Pathological stage					0.769		0.900
	IA	84 (25.7)	59 (25.0)	8 (30.8)		17 (26.2)	
	IB	115 (35.2)	85 (36.0)	7 (26.9)		23 (35.4)	
	IIA	18 (5.5)	13 (5.5)	1 (3.8)		4 (6.2)	
	IIB	33 (10.1)	26 (11.0)	2 (7.7)		5 (7.7)	
	IIIA	66 (20.2)	45 (19.1)	6 (23.1)		15 (23.1)	
	IVA	11 (3.4)	8 (3.4)	2 (7.7)		1 (1.5)	

Thickness					0.759		0.286
	>= 1mm	27 (8.3)	17 (7.2)	2 (7.7)		8 (12.3)	
	< 1mm	300 (91.7)	219 (92.8)	24 (92.3)		57 (87.7)	
TMB							
	Mean	7.64 ± 5.43	7.77 ± 5.58	6.70 ± 4.58	0.349	7.55 ± 5.11	0.778
	Range	0, 42.15	0, 42.15	0, 18.57		0, 20.56	
TMB Level					0.397		0.307
	Low	243 (74.3)	177 (75.0)	22 (84.6)		44 (67.7)	
	High	84 (25.7)	59 (25.0)	4 (15.4)		21 (32.3)	

Categorical data are shown as numbers (%) and continuous data as mean ± SD; TMB, tumour mutation burden.

* the p Value is the test result of the training cohort and the validation cohort;

** the p Value is the test result of the training cohort and the test cohort.
