# Supplementary material for: Predicting response to immunotherapy in advanced non-small-cell lung cancer using tumor mutational burden radiomic biomarker
Source: J Immunother Cancer. 2020 Jul 6;8(2):e000550. doi: 10.1136/jitc-2020-000550 (PMC7342823; doi:10.1136/jitc-2020-000550)
Supplement: Supplementary data [file jitc-2020-000550supp006.pdf]

Table S2 Associations between clinicopathological characteristics and TMB in the training cohort

characteristics	High Level (N=68)	Low Level (N=194)	*p* Value
Age, year	62.7 ± 7.6	61.2 ± 9.3	0.239
Sex			0.595
Male	36 (61.0)	99 (55.9)	
Female	23 (39.0)	78 (44.1)	
Histological subtype			**<0.001**
Adenocarcinoma	10 (16.9)	118 (66.7)	
Squamous cell carcinoma	49 (83.1)	59 (33.3)	
Pathological stage			0.264
IA	13 (22.0)	46 (26.0)	
IB	21 (35.6)	64 (36.2)	
IIA	2 (3.4)	11 (6.0)	
IIB	10 (16.9)	16 (9.0)	
IIIA	13 (22.0)	32 (18.1)	
IVA	-	8 (4.5)	
TMBRB			**<0.001**

Mean	0.63±0.14	0.36±0.12
Range	0.20, 0.82	0.11, 0.78

Categorical data are shown as numbers (%) and continuous data as mean ± SD; TMBRB, tumour mutation burden radiomics biomarker.
