# Supplementary figures and images for: Predicting response to immunotherapy in advanced non-small-cell lung cancer using tumor mutational burden radiomic biomarker
Source: J Immunother Cancer. 2020 Jul 6;8(2):e000550. doi: 10.1136/jitc-2020-000550 (PMC7342823; doi:10.1136/jitc-2020-000550)

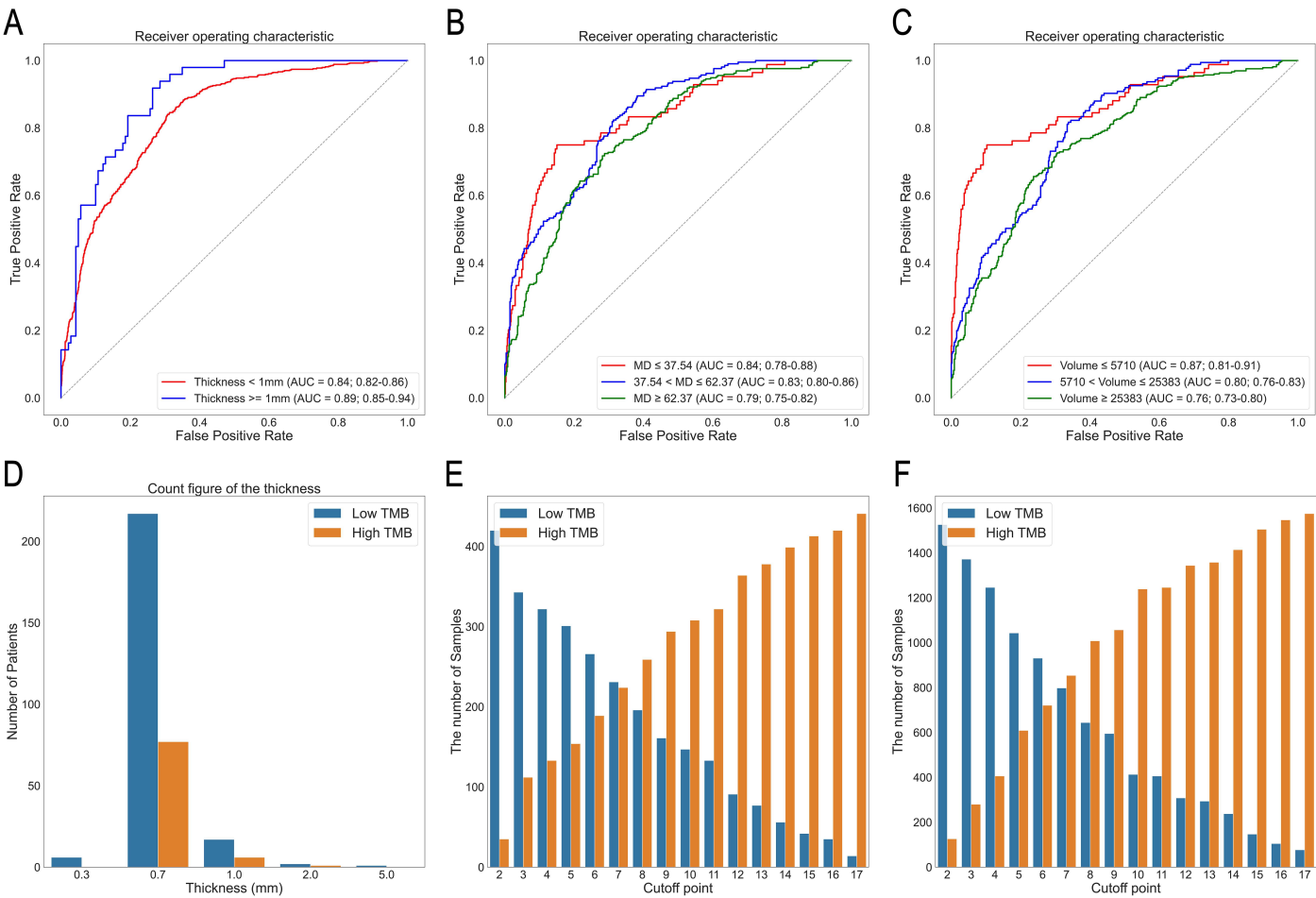

Supplement: Supplementary data [file jitc-2020-000550supp007.pdf]

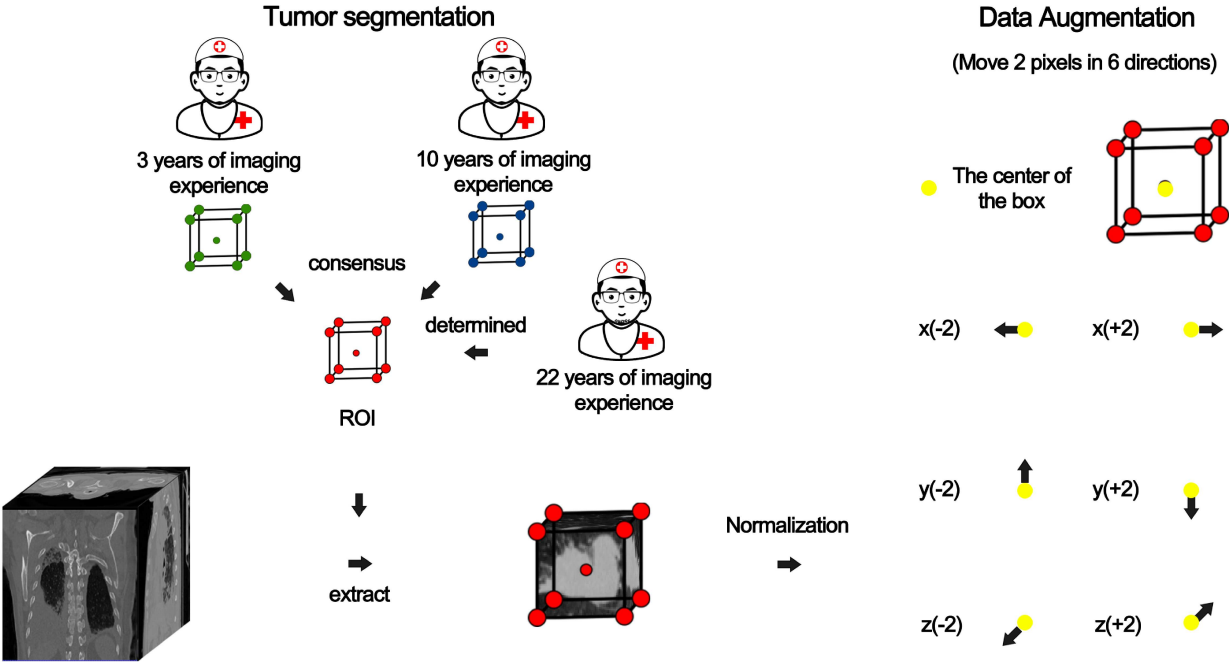

Supplement: Supplementary data [file jitc-2020-000550supp010.pdf]
