# Supplementary material for: Predicting response to immunotherapy in advanced non-small-cell lung cancer using tumor mutational burden radiomic biomarker
Source: J Immunother Cancer. 2020 Jul 6;8(2):e000550. doi: 10.1136/jitc-2020-000550 (PMC7342823; doi:10.1136/jitc-2020-000550)
Supplement: Supplementary data [file jitc-2020-000550supp008.pdf]

Appendix S5: Stratified analysis of TMBRB

Consider whether there are factors that affect TMBRB's judgment and their degree of influence. We included thickness, the maximum three-dimensional diameter (Maximum 3D-diameter) and volume of the tumor as the analysis objects.

For the layer thickness, the data with the thickness of 0.7 mm occupies the highest percentage in the dataset [89.9% (294/327); **Figure S2D**]. Therefore, we chose 1mm as the cut-off to observe the performance of TMBRB for two types of patients. For the tumor volume and the Maximum 3D-diameter, we chose three equal points to divide the dataset into 3 cohorts. Finally, we evaluate TMBRB for all cohorts.

The evaluation results of the TMBRB in hierarchical analysis was shown in **Figure S2**. We found that in the analysis of thickness, we found that both cohorts have shown good results (**Figure S2A**). For the factor of tumor size, regardless of the maximum diameter or tumor volume, the effect of the TMBRB with larger tumors gradually decreases (**Figure S2B and Figure S2C**). We speculated that during resampling, larger tumors are more likely to lose some texture information. In the **Figure S2E** and **Figure S2F**, we show that the closer the TMB cut-off value to TMB median, the better the performance of TMBRB, in particular, for cut-off values within the range 7–9 mut/Mb.

Figure S2. **Results of stratified analysis on the influencing factors of TMBRB**

(A): ROC of TMBRB in cohorts with different thickness; (B): ROC of TMBRB in cohorts with different maximum 3D-diameter; (C): ROC of TMBRB in cohorts with different Volume; (D): The count figure of the thickness in TMB dataset; (E) and (F): Histograms presenting training and validation cohort counts corresponding to different cut-off values.
