# Supplementary material for: Predicting response to immunotherapy in advanced non-small-cell lung cancer using tumor mutational burden radiomic biomarker
Source: J Immunother Cancer. 2020 Jul 6;8(2):e000550. doi: 10.1136/jitc-2020-000550 (PMC7342823; doi:10.1136/jitc-2020-000550)
Supplement: Supplementary data [file jitc-2020-000550supp009.pdf]

Table S3 Clinicopathological characteristics of the immunotherapy dataset

Characteristics	Overall survival			Progression-free survival		
	Hazard Rate (HR)	95%CI	*P* Value	Hazard Rate (HR)	95%CI	*P* Value
Age, year (median as cut-off)	0.88	0.52 ~ 1.47	0.600	1.09	0.72 ~ 1.64	0.700
Sex	0.62	0.34 ~ 1.14	0.100	0.58	0.35 ~ 0.96	**0.030**
Smoking status	0.86	0.50 ~ 1.47	0.600	0.65	0.43 ~ 0.99	**0.040**
ECOG performance-status score	2.38	1.14 ~ 4.96	**0.020**	1.98	1.16 ~ 3.38	**0.010**
Tumor histologic type	0.78	0.46 ~ 1.32	0.300	0.79	0.52 ~ 1.20	0.300
Pathological stage	0.60	0.29 ~ 1.23	0.200	0.67	0.38 ~ 1.19	0.200
EGFR mutations	1.49	0.85 ~ 2.62	0.200	1.47	0.91 ~ 2.38	0.100
TMBRB	0.54	0.31 ~ 0.95	**0.030**	1.78	1.07 ~ 2.95	**0.020**
